# Prediction of adenocarcinoma and squamous carcinoma based on CT perfusion parameters of brain metastases from lung cancer: a pilot study

**DOI:** 10.3389/fonc.2023.1225170

**Published:** 2023-09-20

**Authors:** Chuncheng Jiang, Xin Liu, Qianqian Qu, Zhonghua Jiang, Yunqiang Wang

**Affiliations:** ^1^ Department of Radiology, Yantai Hospital of Traditional Chinese Medicine, Yantai, Shandong, China; ^2^ Department of Oncology, Yantai Hospital of Traditional Chinese Medicine, Yantai, Shandong, China

**Keywords:** CT perfusion, brain metastasis, adenocarcinoma, squamous carcinoma, pathological type

## Abstract

**Objectives:**

Predicting pathological types in patients with adenocarcinoma and squamous carcinoma using CT perfusion imaging parameters based on brain metastasis lesions from lung cancer.

**Methods:**

We retrospectively studied adenocarcinoma and squamous carcinoma patients with brain metastases who received treatment and had been pathologically tested in our hospital from 2019 to 2021. CT perfusion images of the brain were used to segment enhancing tumors and peritumoral edema and to extract CT perfusion parameters. The most relevant perfusion parameters were identified to classify the pathological types. Of the 45 patients in the study cohort (mean age 65.64 ± 10.08 years; M:F = 24:21), 16 were found to have squamous cell carcinoma. Twenty patients were with brain metastases only, and 25 patients were found to have multiple organ metastases in addition to brain metastases. After admission, all patients were subjected to the CT perfusion imaging examination. Differences in CT perfusion parameters between adenocarcinoma and squamous carcinoma were analyzed. The receiver operating characteristic (ROC) curves were used to predict the types of pathology of the patients.

**Results:**

Among the perfusion parameters, cerebral blood flow (CBF) and mean transit time (MTT) were significantly different between the two lung cancers (adenocarcinoma vs. squamous cell carcinoma: *p* < 0.001, *p* = 0.012.). Gender and tumor location were identified as the clinical predictive factors. For the classification of adenocarcinoma and squamous carcinoma, the model combined with CBF and clinical predictive factors showed better performance [area under the curve (AUC): 0.918, 95% confidence interval (CI): 0.797–0.979). The multiple organ metastasis model showed better performance than the brain metastasis alone model in subgroup analyses (AUC: 0.958, 95% CI: 0.794–0.999).

**Conclusion:**

CT perfusion parameter analysis of brain metastases in patients with primary lung cancer could be used to classify adenocarcinoma and squamous carcinoma.

## Introduction

The most prevalent kind of cancer to spread to the brain is lung cancer, which affects 7%–10% of non-small cell lung cancer (NSCLC) patients at diagnosis and 20%–40% of NSCLC patients over time (1–3). Similar to primary lung cancer, the treatment plan for brain metastases should be chosen based on the pathological type. However, primary lung cancer often presents with faint borders and internal necrosis. Furthermore, brain metastases are often tiny and could spread throughout the brain. As a result, invasive biopsy or surgical excision for molecular testing is not always feasible (4). Therefore, developing a noninvasive imaging-based approach to assess the pathological type in patients with brain metastases from lung cancer is advisable.

CT perfusion imaging is a noninvasive functional imaging technique that reflects the hemodynamic changes in tumors with the foundation of enhanced CT (5, 6). In comparison to magnetic resonance imaging (MRI) perfusion imaging, it is insensitive to paramagnetic susceptibility artifacts, is more accessible, has shorter examination times, and has better patient tolerance. Moreover, in clinical practice, we have found that some patients undergo cranial CT scans first due to headaches, which subsequently reveal intracranial metastasis accompanied by lung lesions. Despite the generation of rays during use, CT perfusion imaging can provide higher image resolution and more perfusion parameters than MRI perfusion imaging. Previous studies based on different subtypes of primary lung cancer lesions had shown differences between CT perfusion parameters (7–9). Therefore, CT perfusion analysis is a potentially valuable approach that could be applied to identify pathological types.

However, studies on CT perfusion imaging of lung cancer had mainly focused on primary lung lesions, with fewer relevant studies on brain metastases. Moreover, overcoming or reducing the artifacts caused by respiratory movements had been a problem for us. For these purposes, we extracted perfusion parameters from brain metastasis CT images and aimed to establish a model based on perfusion parameters and clinical features to identify adenocarcinoma and squamous carcinoma.

## Materials and methods

### Patients

**Figure 1:**
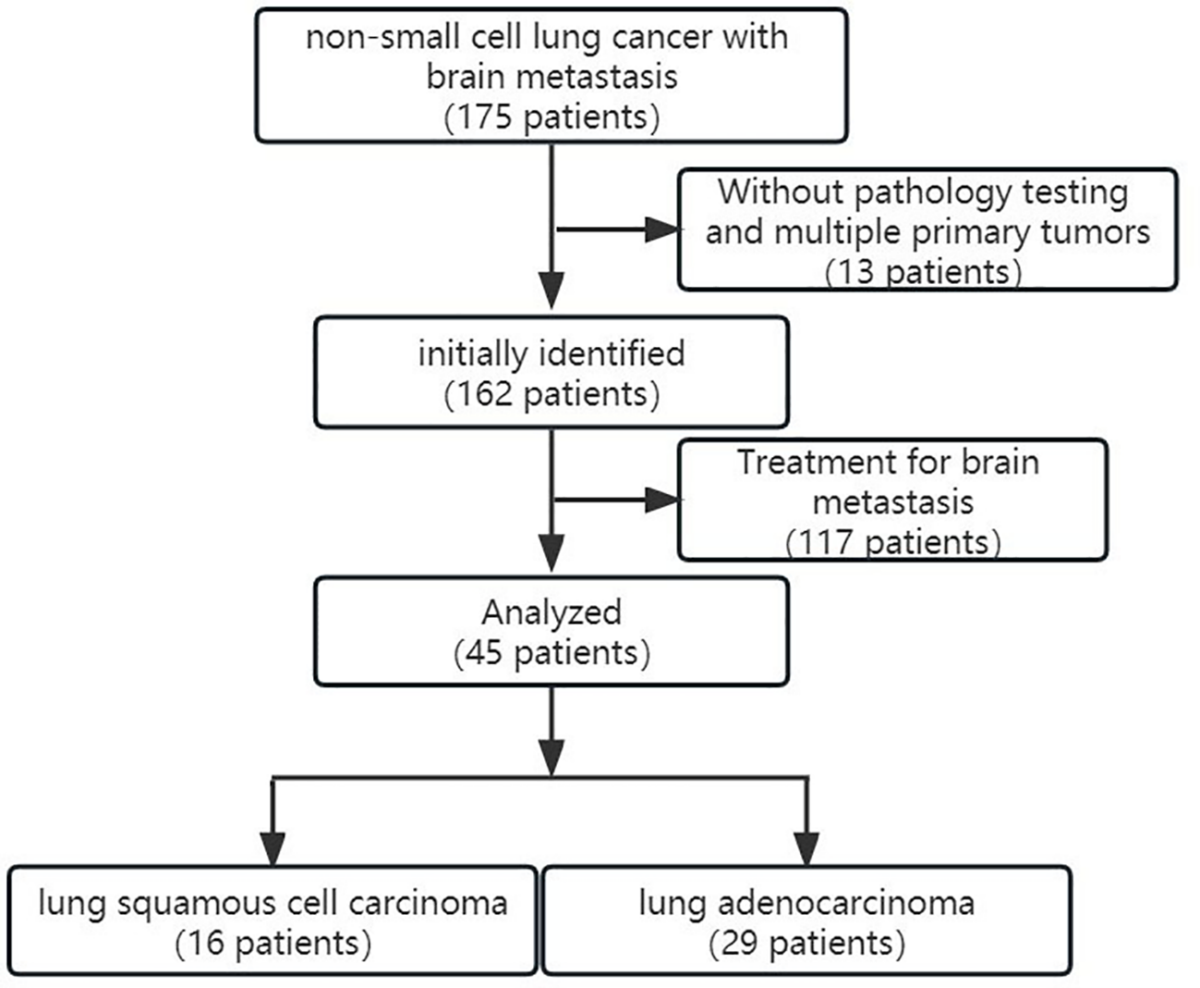
The inclusion and exclusion criteria and the patient enrollment flowchart.

This study was approved by the ethics committee of Yantai Hospital of Traditional Chinese Medicine, Affiliated to Shandong University of Traditional Chinese Medicine. A total of 175 patients underwent CT perfusion examination from December 2019 to December 2021. The inclusion criteria included 1) primary NSCLC metastasis to the brain without a prior history of other tumors, 2) the patient’s pathological type and immunohistochemistry are determined after surgery or biopsy, and 3) no neoadjuvant radiotherapy or chemotherapy specifically targeting brain metastases was performed prior to the CT perfusion examination. The exclusion criteria included 1) multiple primary tumors or other types of lung cancer, 2) no pathological type and immunohistochemical testing was performed, and 3) neoadjuvant radiotherapy or chemotherapy specifically targeting brain metastases was performed before the CT perfusion examination. Finally, 45 patients (mean age 65.64 ± 10.08 years) were enrolled in this study, including 29 cases of lung adenocarcinoma patients and 16 cases of lung squamous cell carcinoma patients (**Figure 1**).

### Pathological type

All patients in this study underwent pathological tests for primary lung cancer. Lung biopsy or lung surgery was used to acquire tissue samples, which were then analyzed using a variety of therapeutically applicable molecular detection technologies, including immunohistochemistry and next-generation sequencing. The study included 29 patients with adenocarcinoma and 16 patients with squamous cell carcinoma.

### CT perfusion image acquisition

We collected the CT perfusion images of lung adenocarcinoma and squamous cell carcinoma patients who were first diagnosed with brain metastasis in our hospital. All patients were scanned by a 128-row 256-slice CT scanner (Brilliance iCT, Philips, Netherlands) for CT perfusion imaging. The high-pressure injector was MEDTRON (Germany). The CT perfusion imaging scheme was Philips jog mode. In this study, 70 mL of contrast agent (iopamidol 370 mgI/mL produced by Beilu Pharmaceutical Co., Ltd.) was injected by a high-pressure syringe through the median elbow vein. Injection flow rate 5 mL/s (4 mL/s for patients with poor vascular elasticity). The CT protocol included 15 spiral acquisitions in succession over the entire tumor before (first acquisition) and after (acquisitions 2–15) the delivery of the contrast material. The total perfusion scanning time was about 61.6 s. The following parameters were used for all acquisitions: tube voltage 80 kV, tube current 100 mAs, collimation 128 × 0.625 mm, and slice thickness 5 mm. The images were reconstructed to 2-mm thickness.

### CT perfusion postprocessing

All images were processed using the Philips CT perfusion software (IntelliSpace Portal v6.0.5.02900). The processing flow was as follows: 1) Browsed all brain images to ensure the integrity of the collected data; 2) Adjusted the position of the mask midline to the brain midline; 3) Selected and adjusted the mask to cover all brain tissues; 4) Removed the mask, and the region of interest (ROI) was selected on the artery and vein (arterial: basilar artery as the reference standard; venous: sagittal sinus/transverse sinus as the reference standard); 5) ROIs were selected on brain metastasis.

### Extraction of CT perfusion parameters

**Figure 2:**
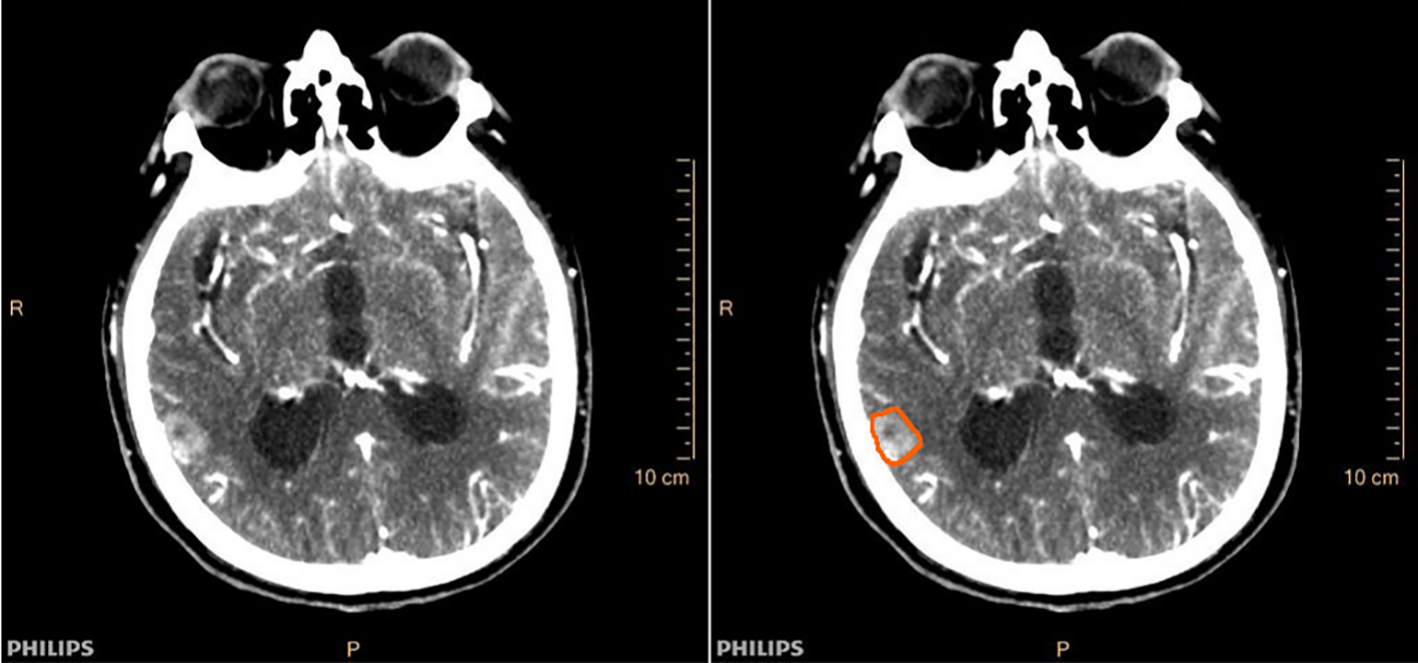
Cranial CT perfusion imaging (left) and region of interest (ROI) outline of intracranial metastases (right) in a patient (male, 78 years old) with brain metastases from lung adenocarcinoma.

Two independent radiologists outlined the ROIs. One intracranial maximal metastasis was selected for each patient, and ROIs were outlined at the largest level of the metastases and its two consecutive layers above and below. Cases with significant discrepancies in certain subjective outlines were reviewed jointly by the supervising physician until an agreement was reached. Finally, the ROIs outlined by one of the radiologists were selected, and perfusion parameters are obtained by machine operations, including cerebral blood volume (CBV), cerebral blood flow (CBF), mean transit time (MTT), and time to peak (TTP). The averages of the three layers of perfusion parameters for each metastasis were used as the final valid parameters. **Figure 2** demonstrated a case of lung adenocarcinoma brain metastasis with CT perfusion images and ROI outlined. To observe the reproducibility of perfusion parameter extraction, we randomly selected 10 patients to perform the intraclass correlation coefficient (ICC) test on the ROI outlined by two radiologists, and ICC ≥0.8 was considered as better reproducibility.

### Data analysis

Statistical analysis was performed with SPSS (version 23.0); GraphPad Prism (version 8.0.2). The independent-sample t-test or Mann–Whitney U test was used to compare perfusion parameters. Measurement data were expressed as $\bar{\text{x}}$ ± s. The count data were expressed as n (%), and the chi-square test or Fisher’s exact test was used. The ROC and AUC were used to assess the predicted energy efficiency of CT perfusion parameters. The DeLong test was used to compare the differences between different prediction models. *p* < 0.05 was considered statistically significant.

## Results

### Clinical features

**Table 1** summarized the clinicopathological characteristics of 45 patients from the hospital archive electronic information system. Statistical analysis showed that age, smoking, metastasis number, maximum diameter of metastases, maximum diameter of tumor, T staging, N staging, and treatment were not significantly correlated with the pathological type. However, there were significant differences between adenocarcinoma and squamous carcinoma patients in terms of gender and location of the tumor (*p* = 0.005, *p* = 0.017).

**Table 1:** The clinicopathological characteristics of the 45 patients.

Characteristics	Type of lung cancer		*p*
	Lung adenocarcinoma (n =29)	Lung squamous cell carcinoma (n =16)	
Age, years^a^	65.24±10.25	66.38±10.04	0.722
Metastasis maximum diameter,cm^a^	1.45±0.31	1.52±0.27	0.704
Metastasis number,n(%)			0.509
1≤n≤3	21(72.41%)	13(81.25%)	
n>3	8(27.59%)	3(18.75%)	
Sex, n (%)			0.005^*^
Male	11(37.93%)	13(81.25%)	
Female	18(62.07%)	3(18.75%)	
Smoking, n (%)			0.373
Yes	13(44.83%)	11(68.75%)	
No	16(55.17%)	5(31.25%)	
Tumor maximum diameter,cm^a^	5.02±1.42	5.79±1.27	0.075
Location of tumor n (%)			0.017^*^
Central	11(37.93%)	12(75.00%)	
Peripheral	18(62.07%)	4(25.00%)	
T staging n (%)			0.492
T1+T2	6(20.69%)	2(12.50%)	
T3+T4	23(79.31%)	14(87.50%)	
N staging n (%)			0.372
N0+N1	9(31.03%)	3(18.75%)	
N2+N3	20(68.97%)	13(81.25%)	
Treatment n (%)			
None	22(75.86%)	13(81.25%)	0.561
Radiotherapy and/or Chemotherapy	5(17.24%)	3(18.75%)	
Targeted therapies	2(6.90%)	0	

a Mean ± SD.

*p < 0.05.

### Classification of NSCLC

**Figure 3:**
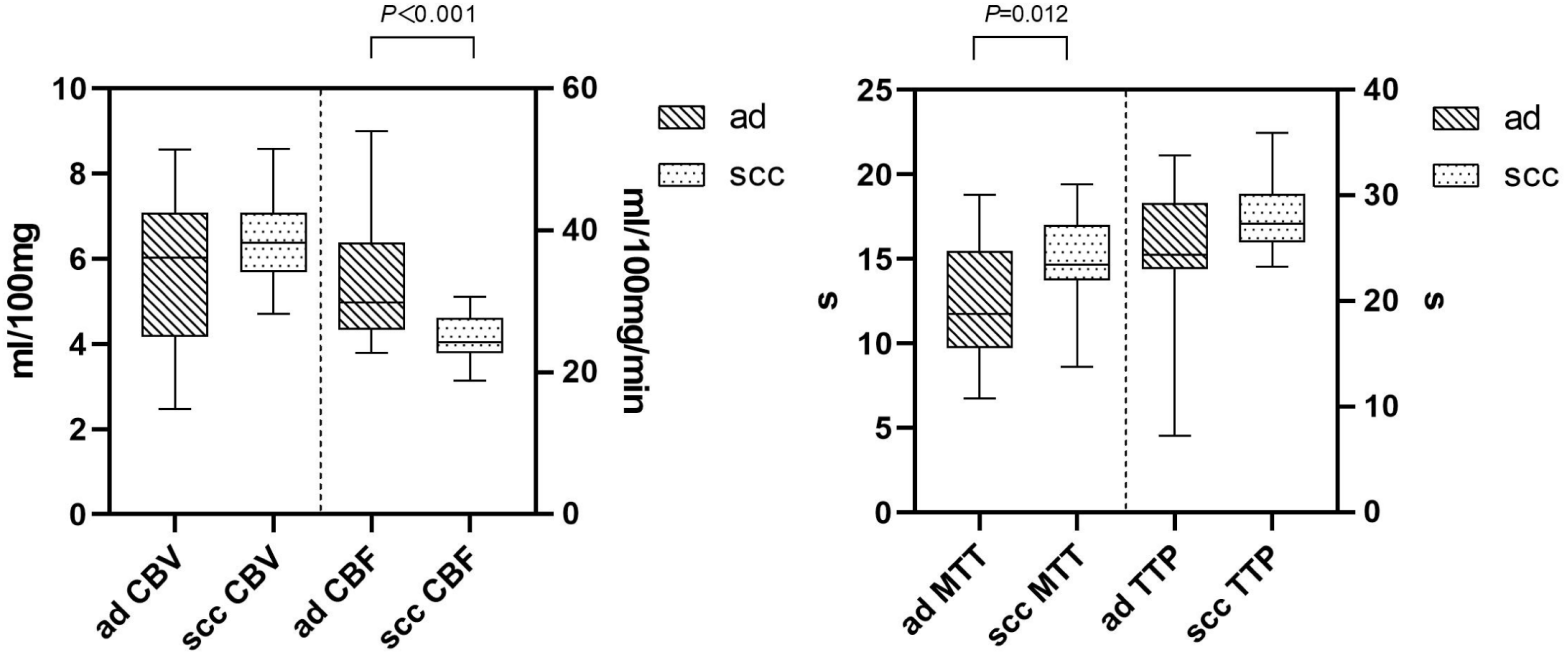
Comparison in CT perfusion parameters of brain metastases. The y-axis represents the unit, while the x-axis represents the perfusion parameters. The CBF of the brain metastasis in adenocarcinoma was significantly higher than that of squamous cell carcinoma (left). CBV stands for cerebral blood volume, and its unit is mL/100 mg. CBF stands for cerebral blood flow, and its unit is mL/100 mg/min. The MTT of the brain metastasis in adenocarcinoma was significantly lower than that of squamous cell carcinoma (right). MTT stands for mean transit time, and its unit is seconds (s). TTP stands for time to peak, and its unit is seconds (s). “ad” stands for adenocarcinoma of the lung, while “scc” stands for squamous cell carcinoma of the lung.

**Figure 4:**
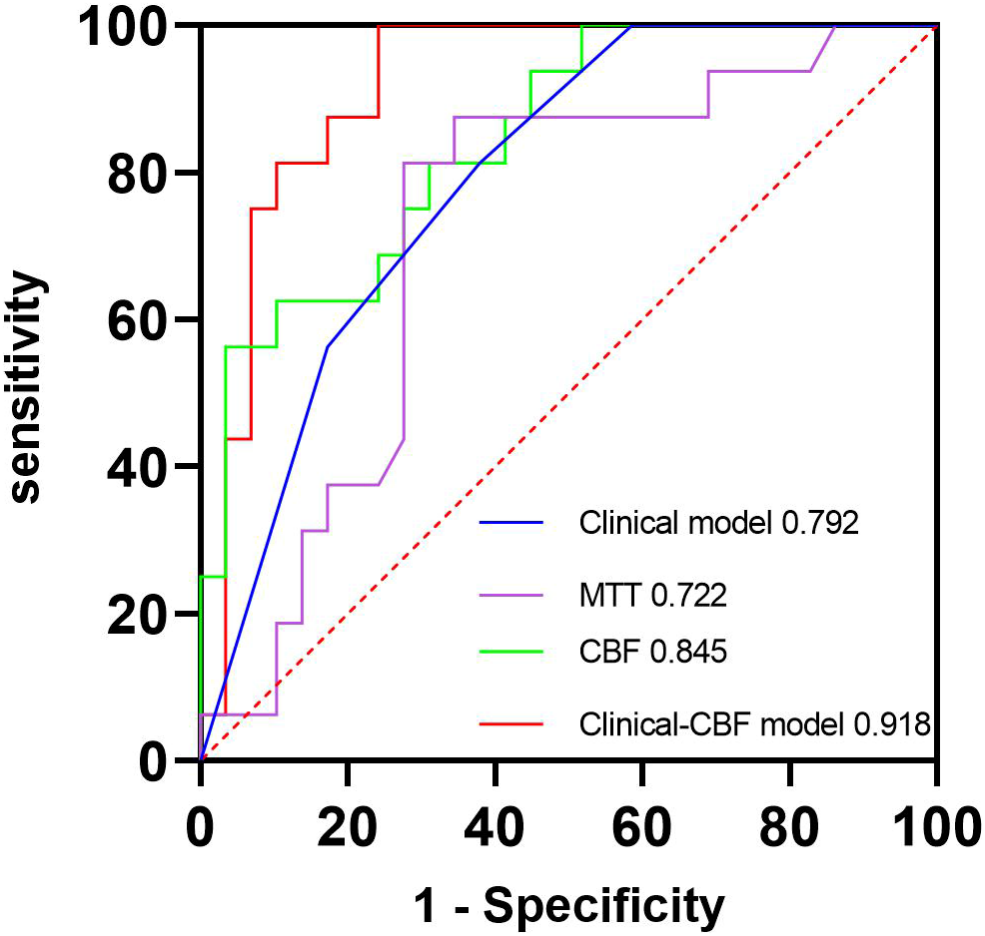
ROC curves of the clinical model, MTT, CBF, and clinical-CBF model. The blue line represents the clinical model, and its AUC is 0.792. The purple line represents the MTT parameter model, and its AUC is 0.722. The green line represents the CBF parameter model, and its AUC is 0.845. The red line represents the clinical combined with CBF model, and its AUC is 0.918. AUC, area under the curve; CBF, cerebral blood flow; MTT, mean transit time; ROC, receiver operating characteristic.

**Figure 5:**
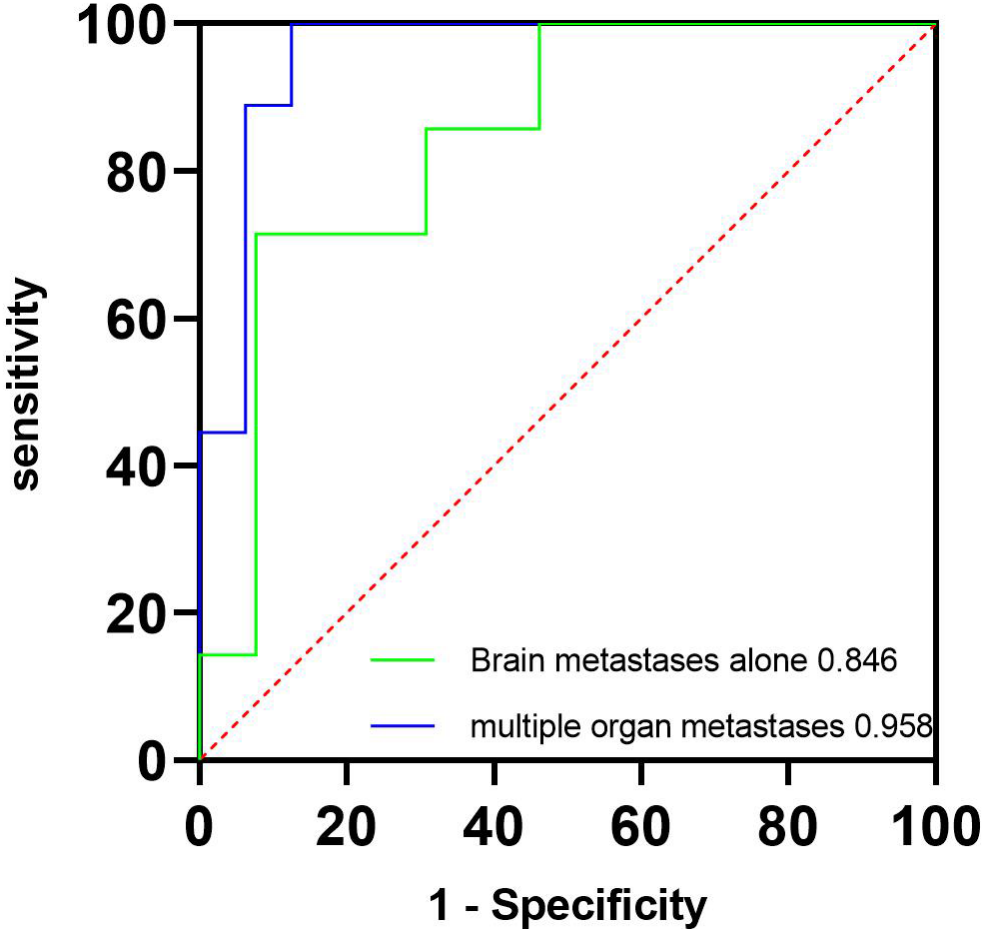
ROC curves of the multiple organ metastasis model and the brain metastasis alone model. The green line represents the brain metastasis alone model, and its AUC is 0.846. The blue line represents the multiple organ metastasis model, and its AUC is 0.958. AUC, area under the curve; ROC, receiver operating characteristic.

The ICC of perfusion parameter extraction had achieved 0.83 between the two different radiologists. The CBF of brain metastasis in adenocarcinoma was significantly higher than that of squamous cell carcinoma (*p* < 0.001, Mann–Whitney U test), while the MTT of brain metastasis in adenocarcinoma was significantly lower (*p* = 0.012, independent-sample t-test) (**Figure 3**). The ROC curve analysis showed that the AUCs of CBF, MTT, clinical model, and clinical-CBF model in predicting pathological types were 0.845 [95% confidence interval (CI): 0.706–0.935], 0.722 (95% CI: 0.568–0.845), 0.792 (95% CI: 0.645–0.898), and 0.918 (95% CI: 0.797–0.979), respectively (**Table 2**, **Figure 4**). The DeLong test showed that there was a significant difference between the clinical-CBF model and MTT, clinical model (*p* = 0.017, *p* = 0.018) but showed no significant difference between the clinical-CBF model and CBF (*p* = 0.112). In subgroup analyses, the AUCs of the multiple organ metastasis model and the brain metastasis alone model were 0.958 (95% CI: 0.794–0.999) and 0.846 (95% CI: 0.617–0.966) (**Table 3**, **Figure 5**).

**Table 2:** Predictive performance of the four models.

	AUC (95% CI)	Sensitivity	Specificity
CBF	0.845 (0.706 - 0.935)	0.563	0.966
MTT	0.722 (0.568 - 0.845)	0.813	0.724
Clinical model	0.792 (0.645 - 0.898)	0.813	0.621
Clinical-CBF model	0.918 (0.797 - 0.979)	1.000	0.759

AUC, area under the curve; CBF, cerebral blood flow; CI, confidence interval; MTT, mean transit time.

**Table 3:** Predictive performance of brain metastasis alone and multiple organ metastasis models.

	AUC (95% CI)	Sensitivity	Specificity
Brain metastasis alone model	0.846 (0.617 - 0.966)	0.714	0.923
Multiple organ metastasis model	0.958 (0.794 - 0.999)	1.000	0.875

AUC, area under the curve; CI, confidence interval.

## Discussion

In this study, we collected perfusion parameters from CT images of brain metastatic lesions. Subsequently, we used the perfusion parameters to build models for the classification of pathological types of the two most common cancers in NSCLC patients, namely, adenocarcinoma and squamous cell carcinoma. Our findings suggested that CT imaging-based perfusion parameters of brain metastases could be used as a noninvasive tool to distinguish pathological types of NSCLC.

Currently, the pathologic type of NSCLC remains an important basis for patient treatment and prognosis. With the progress of lung cancer treatment and research, some therapies and drugs, such as immune checkpoint inhibitors and targeted therapies, had shown better results in providing longer survival and better quality of life for patients with brain metastases of lung cancer. The prerequisite for these precise treatments is the clarification of the pathologic type.

Our study originally used a perfusion parameter approach to analyze CT images of brain metastatic lesions to classify adenocarcinoma and squamous carcinoma, whereas previous studies concentrated on using perfusion parameters of primary cancers. For example, Tacelli et al. (10) proved that CT perfusion imaging technology may, to some extent, reflect the creation of pulmonary capillaries and the ability of tumor metastasis and dispersion. Bevilacqua et al. (8) found that the BF of adenocarcinoma was significantly higher than that of squamous cell carcinoma, which may be used to guide treatment strategy. Chen et al. (7) used perfusion parameters from low-dose CT images of primary lesions to differentiate adenocarcinoma and squamous cell carcinoma. However, no significant difference was found.

CT perfusion imaging plays an important role in evaluating patients with acute ischemic stroke (11–13). And there are still many challenges in diagnosing brain tumors. However, we believe that there are still clues to follow in distinguishing between intracranial metastases and intracranial primary tumors. First, CT perfusion imaging is based on contrast-enhanced CT scans, which can easily distinguish brain metastases that are more commonly located in the cerebral cortex and the cortical–subcortical junction. Second, brain metastases not only have a history of primary lung cancer but also exhibit characteristics such as small nodular lesions and significant edema, which can be easily differentiated on CT perfusion images. Recent research had shown that perfusion imaging has the ability to differentiate between brain metastases and intracranial primary tumors (14). Our CT perfusion imaging of brain metastases was based on two considerations. On one hand, primary lesions and metastases showed high consistency in pathological type. On the other hand, we believed that this approach can avoid lung breathing artifacts on perfusion parameters. Despite the fact that the pathology samples of the patients were collected from primary lung cancer, our CT perfusion analysis of brain metastases should still have merit. At first, approximately 10% of NSCLC patients have brain metastases diagnosed before lung cancer (15). A CT perfusion examination of their brain metastases prior to the primary lung cancer biopsy or resection specimen may provide significant information on the pathological type of their primary lung cancer. Second, in actual practice, the pathology of lung cancer may not always be available (16). In contrast to invasive biopsy or surgery for either the original lung cancer or the brain metastases, brain CT perfusion scans are noninvasive and considerably easier to get. Third, in contrast to relying merely on primary lung cancer to predict the pathological type, the CT perfusion analysis of the brain metastases not only avoids the interference of respiratory movement but also serves as a helpful addition.

Our results were not directly comparable to previous correlative studies due to different perfusion approaches. However, some similar perfusion parameters could be used for reference, such as our study found that the CBF of adenocarcinoma was significantly higher than that of squamous cell carcinoma, which was similar to the study by Bevilacqua et al. (8) and suggested that adenocarcinoma patients would most benefit from antiangiogenic therapies. Our subgroup study showed that the multiple organ metastasis model exhibited a higher AUC than the brain metastasis alone model, which we believed may be due to the fact that multiple organ metastases imply a greater invasive capacity of the tumor and higher blood flow. Although adding clinical factors, our highest AUC for predicting pathological types showed no significant difference with the CBF model (0.918 vs. 0.845, *p* = 0.112), which may be caused by the small sample size. We believed that further studies with a larger sample size will surely make the prediction model more reliable.

Several limitations of our research should be noted. First, this study was retrospective, and we collected CT perfusion parameters from lung cancer brain metastases. Some confounding variables could not be sufficiently controlled. For example, patients who had good vascular elasticity arranged the 5 mL/s flow rate of contrast administration, while those with poor vascular elasticity patients arranged 4 mL/s. Furthermore, patients in our research cohort may have received different treatments for primary lung cancer before brain metastases. We lacked the statistical capacity to account for the impact of various treatment regimens on predictive modeling. Second, our results were based on a limited number of samples in a single center, and our models had not been validated with external data, which may lead to bias; therefore, conclusions driven by the potential predictive value of CT parameters should be taken with caution. Third, we delineated ROIs with larger brain metastases for the reason that tiny or cystic metastases may lead to unreliable results. Nevertheless, the results reported in this paper may be used as preliminary data to support prospective studies using CT perfusion parameters to classify pathological types in patients with brain metastases.

In summary, our study showed that CT perfusion parameters based on brain metastasis can be used as a noninvasive approach to classify adenocarcinoma and squamous carcinoma. To validate our findings, future research should be carried out with a larger sample size, multicenter, and multiple clinical features.

## Data availability statement

The raw data supporting the conclusions of this article will be made available by the authors, without undue reservation.

## Ethics statement

The studies involving humans were approved by ethical committee of Yantai Hospital of Traditional Chinese Medicine Yantai Hospital of Traditional Chinese Medicine. The studies were conducted in accordance with the local legislation and institutional requirements. The ethics committee/institutional review board waived the requirement of written informed consent for participation from the participants or the participants’ legal guardians/next of kin because accordance with the national legislation and the institutional requirements.

## Author contributions

CJ, ZJ, and YW: study design. CJ, XL, and QQ: data collection. CJ, XL, and QQ: data processing. CJ and XL: manuscript writing. YW, ZJ: funding acquisition. All authors contributed to the article and approved the submitted version.
